# Treatment strategies in MDA5-positive clinically amyopathic dermatomyositis: a single-center retrospective analysis

**DOI:** 10.1007/s10238-024-01300-8

**Published:** 2024-02-17

**Authors:** Stefanie Hirsch, Gesa Helen Pöhler, Benjamin Seeliger, Antje Prasse, Torsten Witte, Thea Thiele

**Affiliations:** 1 Department of Rheumatology and Immunology, Hannover Medical School, Carl-Neuberg-Str. 1, 30625 Hannover, Germany; 2 Department of Diagnostic and Interventional Radiology, Hannover Medical School, Hannover, Germany; 3 Department of Pneumology and Infectious Diseases, Hannover Medical School, Hannover, Germany

**Keywords:** MDA5, Dermatomyositis, Interstitial lung disease (ILD), Treatment strategies

## Abstract

Melanoma differentiation-associated protein 5 (MDA5) antibody positive amyopathic dermatomyositis (DM) is a rare inflammatory disease. So far, there is no official treatment guideline in MDA5 amyopathic dermatomyositis, but early and aggressive immunosuppressive combination treatment can induce a stable remission. We retrospectively analyzed a cohort of eight patients (male *n* = 5) that were diagnosed with MDA5-positive amyopathic DM. Patient data comprised demographics, CT-guided diagnosis of pulmonary involvement, pulmonary function testing including forced vital capacity (FVC) and diffusing capacity for carbon monoxide (DLCO) data on baseline and mean long-term follow-up of 51 months (24–92 months) to evaluate treatment strategies. Depending on severity of organ involvement treatments were individualized including cyclophosphamide, immunoglobulins and plasmapheresis. Simultaneously, oral treatment with tacrolimus was commenced in four of the eight patients. Most patients received remission maintenance therapy with a combination of tacrolimus, rituximab and low dose steroids. In all patients, improvement in FVC was recorded and five patients achieved an improvement in DLCO. An improvement in the CT imaging morphological findings was observed in four patients. Awareness for the entirety of all clinical and disease-related findings of amyopathic DM is crucial, and remission maintenance is often achieved with a combination of tacrolimus and rituximab.

## Introduction

Clinically amyopathic dermatomyositis (CADM) is a serious and potentially life-threatening variant of dermatomyositis (DM) characterized by typical skin manifestations and severe and rapidly progressive pulmonary involvement in the absence of muscle inflammation or weakness and elevated CK. The anti-melanoma differentiation-associated gene 5 (anti-MDA5) antibody is associated with CADM and is used for its early diagnosis. CADM is more frequent in Asia (up to 35% of DM) than in Europe (7–15% of DM). As a consequence the published data mostly refer to Asian patients with a MDA5-DM [1].

The complication of rapidly progressive interstitial lung disease (RPILD) is still associated with a high mortality [2]. Up to 80% of CADM anti-MDA5 with RPILD patients do not survive even after an early diagnosis and intensive immunosuppressive therapy [3].

So far, there are no treatment guidelines for this disease. Glucocorticoids, azathioprine, methotrexate, intravenous immunoglobulins, cyclophosphamide, calcineurin inhibitors or rituximab have been suggested to be of therapeutic benefit based on case series and a multicenter prospective study of the efficacy and safety of combined immunosuppressive therapy in MDA5-DM [4–9]. Active malignant disease concurrent to CADM makes therapeutic decisions even more challenging, our cohort includes two patients that were diagnosed and treated in this particular setting.

It is still unclear, how long immunosuppressive therapy should be continued. Here, we describe our experiences with long-term treatment in eight patients with CADM.

Immune checkpoint inhibitors (ICI) that are increasingly used in cancer treatments are also known to induce ICI-related inflammatory diseases as adverse events in a significant amount of patients including dermatomyositis and polymyositis like diseases. Even though our cohort does not include patients with ICI-treatment, better understanding of disease course and individual treatment of CADM might help to improve the treatment of other variants of DM as well [10].

## Patients, materials and methods

### Patient cohort and acquisition of clinical data

All patients with MDA5-positive DM visiting our rheumatology department for in- or outpatient treatment from January 2017–September 2021 were included. The data of positive MDA5 antibodies were requested from our laboratory.

### Consent to participate

Written informed consent was obtained from all individual participants included in the study. This data analysis was retrospectively achieved and was approved by the Local Ethics Committee of Hannover Medical School.

### Assessment

Information including patients` age, sex, symptoms at first onset, pattern of pulmonary involvement, pulmonary function test and comorbidities were systematically evaluated from patients’ files and interviews and documented.

Previous and ongoing therapies performed in our department and in external medical facilities were documented.

### Laboratory studies

The MDA5 antibody test was performed along with 16 other myositis-associated antibodies using EUROLINE Autoimmune Inflammatory Myopathies 16 Ag (Euroimmun, Luebeck, Germany). Additional antibodies were tested (ANA, ENA, double-stranded DNA antibodies) at least at the first patients’ visit. Furthermore, we performed our standard laboratory testing including whole blood count, C-reactive protein (CRP), erythrocyte sedimentation rate (ESR), lactate dehydrogenase (LDH), ferritin and creatinine kinase (CK) at the first and every follow-up visit.

## Results

During the observation period, MDA5 antibodies were detected in eight patients treated in our department. In two patients, MDA5 had already been externally identified prior to the referral to our clinic and confirmed in our laboratory. All patients fulfilled criteria for rapidly progressive CADM-ILD. Rapidly progressive CADM-ILD is characterized by the onset of pulmonary symptoms within three month from beginning of initial presentation [11, 12].

Three of the eight patients were female. Mean age at diagnosis was 48.5 years (range 13–79 years). Mean duration of treatment in our department is 51 months (24–92 months).

The most common symptoms at first onset were dyspnea (three patients) with one patient requiring oxygen supplementation, weight loss (five patients), myalgia (four patients), arthralgia (three patients) and fatigue (one patient). Skin manifestations occurred in five patients (calcinosis, heliotrope rash, Gottron papules, and mechanic`s hands). All patients characteristics are shown in Table 1.

**Table 1:** Demographic and laboratory findings

	P1	P2	P3	P4	P5	P6	P7	P8
Sex	f	m	f	f	m	m	m	m
Age at diagnosis	79	48	36	55	68	13	32	57
Treatment period (in months)	49	66	67	53	32	92	29	24
Symptoms at onset	Skin changings, weight loss, night sweats, arthralgias	Skin changings, weight loss, progressive dyspnea	Gottron papules, mechanic hands, myalgias	Heliotrope rash, progressive dyspnea, weight loss, myalgias	Fatigue, weight loss, arthralgias	Calcinosis, arthralgias	Dyspnea, weight loss, myalgias, arthritis	Myalgias, arthalgias, thoracic pain
CK (creatine kinase) at first presentation (U/L) (< 145 U/L)	36	29	17	66	20	126	78	36
C-reactive protein at first presentation (mg/L) (< 5 mg/L)	18.2	36.6	6.1	11.9	16.5	2.6	0.7	4.9
Sedimentation rate (ESR) at first presentation	5/8	n.a	n.a	n.a	39/75	14/30	32/71	26/59
LDH (Lactate dehydrogenase) at first presentation (U/L) (< 247)	379	590	232	233	253	221	386	184
Ferritin (µg/L) at first presentation (27–365)	579	1862	875	351	880	n.a	458	1295
Other antibodies	ANA (1:160 spec), DNA-Crithidia	ANA (1:640 spec), Ro52	ANA (1:1280 sp/zyto), SS-A/Ro	ANA (1:320 spec), SS-A/Ro, Ro52	ANA (1:2560 ho/sp), rheumatoid factor	ANA (1:80), rheumatoid factor, CCP-AK	ANA (1:80), rheumatoid factor, Cardiolipin IgM, SS-A/Ro	ANA (1:80)

### Laboratory findings

CK was in the normal range of our laboratory in every patient of this cohort at first presentation. C-reactive protein was elevated in five out of eight patients (mean 12.2 mg/L (0.7–36.6 mg/L). LDH was elevated in three patients. Ferritin was elevated in six out of eight patients (mean 900 µg/L (351–1862 µg/L)).

In the screening procedure, the following additional autoantibodies were identified: rheumatoid factor (two patients), ACPA (anti-citrullinated peptide) (one patient), ANA with a titer > 1:160 (seven patients), SS-A-antibodies (three patients), of these two with Ro52 antibodies.

### Pulmonary involvement and pulmonary function testing

All eight patients had changes in the initial computed tomography of the chest (CT chest) at diagnosis. Three patients had basally emphasized interstitial lung disease, two patients had ground-glass opacities and another patient had a basal interstitial pneumonia (Table 2). In two patients, extensive interstitial lung parenchyma changes were described. In one patient, cicatricial contortions of the lung parenchyma were described, which were not typical for a lung fibrosis.

**Table 2:** Radiographic findings

	Radiological findings at diagnosis	Radiological findings at 1st follow-up	Days to 1st follow-up	Radiological findings at 2nd follow-up	Days to 2nd follow-up
P1	Interstitial parenchyma changes, bihilary lymphadenopathy	Regression	231	Indurative lung parenchyma changes	395
P2	Extensive interstitial lung parenchyma changings	Regression	376	Minimal ground-glass opacity	845
P3	Basal emphasized interstitial lung disease	Regression	1579	n.a	n.a
P4	Basal emphasized interstitial lung disease	Unchanged	1160	n.a	n.a
P5	Basal emphasized interstitial lung disease	Unchanged	270	n.a	n.a
P6	Basal interstitial changing	Unchanged	589	No interstitial changing, basal pitted fibrosis	3009
P7	Extensive interstitial lung parenchyma changings	Regression	246	n.a	n.a
P8	Cicatricial contortions of the lung parenchyma	Unchanged	353	n.a	n.a

A first follow-up CT of the chest was performed in eight patients after 600 days on average (231–1579 days). There were stable findings in four patients.

In four patients, an improvement of the initial radiological findings in the baseline CT was described. A second follow-up CT of the chest was performed in three patients (P1, P2 and P6) with no further changes compared to the first follow-up.

All patients received pulmonary function testing at first presentation (Table 3). During the course of treatment, there was an improvement in six patients in DLCO overall from 50% (25–79%) to 63% (40–97%). In one patient, DLCO remained stable and in another patient there was a decrease in DLCO (P8).

**Table 3:** Pulmonary function testing

	DLCO at diagnosis (%)	FVCex at diagnosis (%)	DLCO at follow-up (%)	FVC ex at follow-up (%)	days to follow-up
P1	42	108	68	110	173
P2	25	47	51	67	1184
P3	61	69	73	70	735
P4	53	85	52	109	530
P5	46	46	55	50	542
P6	57	47	97	81	1770
P7	34	38	40	47	179
P8	79	67	68	75	333

In all patients, an improvement was measured in FVCex (Forced expiratory Capacity) during treatment. At diagnosis, mean FVCex was 63.4% (38–108%) and at follow-up 76.1% (47–110%). Follow-up was performed after 680 days on average (173–1184 days).

### Bronchoscopy and bronchoalveolar lavage

Bronchoscopy and bronchoalveolar lavage (BAL) was performed in three patients.

The findings differ. There was a normal finding in the bronchoscopy and a mild neutrophilia in the BAL in one patient. A bronchitis (bronchoscopy) with lymphocytosis and neutrophilia (BAL) was shown in another patient. The third bronchoscopy showed a chronic bronchitis with severe lymphocytosis and neutrophilia (BAL).

### Treatment strategies

Individual treatments are listed in Table 4.

**Table 4:** Treatment strategies

Remission induction	Remission maintenance
Chemotherapy with R + CHOP by diagnosis of a non-Hodgkin lymphoma (R; Rituximab, C; Cyclophosphamide, H; Doxorubicin, O; Vincristine, P; Prednisone)	Prednisone 1.5 mg per day
Steroid pulse (1000 mg per day for 3 days) followed by oral Prednisone 70 mg/day with tapering schedule + parallel plasmapharesis (7x) + parallel initiation Cyclophosphamide (every four weeks, for 6 times) + parallel intravenous immunoglobuline administration ( every four weeks, for 6 times (95 g each over 3–4 days) + parallel initiation Tacrolimus 9 mg 1–0–1 per day	Tacrolimus 7.5 mg 1–0–1 (Target serum level: 10 µg/L)
Steroid pulse followed by oral Prednisone with tapering schedule + parallel Mycophenolate mofetil switch Azathioprine and Hydroxychloroquine + Tacrolimus topically	Azathioprine 50 mg 1–0–1 Hydroxychloroquine 200 mg 1–0–0 Tacrolimus topically
oral Prednisone 50 mg/day with tapering schedule + parallel initiation Cyclophosphamide (every four weeks, for 6 times) + prostaglandine intravenous + parallel initiation Tacrolimus 4 mg 1–0–1 per day	Tacrolimus 4 mg 1–0–1 (Target serum level: 10 µg/L) Prednisone 4 mg per day
Steroid pulse (500 mg per day for 3 days) followed by oral Prednisone 80 mg/day with tapering schedule + parallel plasmapharesis (5x) + parallel initiation Cyclophosphamide (every four weeks, for 6 times) + parallel initiation Tacrolimus 2 mg 1–0–1 per day	Tacrolimus 2.5 mg 1–0–1 Prednisone 5 mg per day Rituximab 1000 mg every six month
Steroid pulses (2015–2018) followed by Mycophenolate mofetil followed by Tocilizumab followed by Cyclosporine A	Cyclosporine A 150 mg 1–0–1
Oral Prednisone and Methotrexate switch Methotrexate + Azathioprine + Hydroxychloroquine followed by oral Prednisone + prostaglandine intravenous + intravenous immunoglobuline (Proof of MDA5) Dose increase oral Prednisone 40 mg/day with tapering schedule + parallel initiation Cyclophosphamide (every four weeks, for 6 times) followed by R ituximab (every six month) + parallel initiation Tacrolimus 2 mg 1–0–1 per day	Tacrolimus 2 mg 1–0–1 (Target serum level: 10 µg/L) Hydroxychloroquine 200 mg 1–0–1 Prednisone 5 mg per day Rituximab 1000 mg every six month
Chemotherapy with Dnr/AraC/Midostaurin by diagnosis of acute myeloid leukemia (Dnr; Daunorubicin, AraC; Cytarabin) (Proof of MDA5) Recommendation to start Tacrolimus orally, patient denied	Watch and wait

In patient 1 (P1), a non-Hodgkin lymphoma was diagnosed concurrently to the diagnosis of the MDA5-positive DM and treated with RCHOP-chemotherapy regimen. Currently, P1 is in remission on a treatment with 1.5 mg prednisone per day.

Due to severe pulmonary involvement, patient 2 (P2) required oxygen supplementation at initial diagnosis and treatment at our rheumatology ward. Therapeutically, an intravenous steroid pulse with 1000 mg prednisone per day for three days followed by tapering oral glucocorticoids was initiated in combination with seven cycles of plasmapheresis. This induction therapy was followed by intravenous immunoglobulins and cyclophosphamide bolus therapy for overall 6 month. P2 obtained 90-g intravenous immunoglobulins every four weeks. Cyclophosphamide was administered first with 500 mg/m^2^, after four weeks 750 mg/m^2^, followed by 1000 mg/m^2^ every four weeks for a total of six infusions. In case of reduced leukocytes, ten days after the cyclophosphamide infusion the increase of the dose was suspended. An additional immunosuppressive therapy with oral tacrolimus was initiated (target serum level 10 µg/L) parallel to the first cycle of cyclophosphamide due to the disease severity and rapid progression of the interstitial pneumonia. The patient reached stable disease remission after 24 weeks and remains on a steroid-free maintenance therapy with tacrolimus (7.5 mg twice daily).

In patient 3 (P3), therapy was initially started with high-dose glucocorticoids and mycophenolate mofetil, followed by combination treatment of glucocorticoids, azathioprine and hydroxychloroquine. Tacrolimus was applied topically. This therapy is still proceeded.

Patient 4 (P4) therapy was initiated with oral prednisone (1 mg/KG/kg) per day with tapering schedule. In parallel, cyclophosphamide was administered with 500 mg/m^2^, after four weeks 750 mg/m^2^, followed by 1000 mg/m^2^ every four weeks for a total of six infusions, and oral tacrolimus was initiated. For remission maintenance, only tacrolimus (4 mg twice daily) is currently necessary.

In patient 5 (P5), intravenous steroid pulse with 500 mg prednisone per day for three days was performed, followed by oral glucocorticoids with a tapering schedule. P5 received five cycles of plasmapheresis during the first stay in our hospital. Cyclophosphamide first was administered with 500 mg/m^2^, after four weeks 750 mg/m^2^, followed by 1000 mg/m^2^ every four weeks for a total of six infusions. And in parallel, oral tacrolimus orally was commenced. Currently, P5 obtains 2.5 mg of tacrolimus twice daily, and rituximab was started with a dose of 1000 mg every six months to maintain remission.

In patient 6 (P6), initially seronegative rheumatoid arthritis or undifferentiated connective tissue disease was suspected. Steroid pulses were performed, followed by a treatment with tocilizumab (anti-IL-6R antibody). After the detection of MDA5 antibodies and progressive disease in 2015, the treatment was switched to oral cyclosporine. Glucocorticoids were discontinued in 2019.

In patient 7 (P7), the diagnosis was unclear at the first onset of symptoms. P7 was treated with prednisone and methotrexate, followed by methotrexate plus azathioprine and hydroxychloroquine. When P7 deteriorated rapidly with extensive interstitial lung parenchyma changings, he received intravenous prostaglandins and immunoglobulins and was referred to our hospital. After the detection of MDA5 antibodies, the daily dose of prednisone was increased (40 mg/per day) and tapered subsequently, and in parallel, intravenous cyclophosphamide was administered with 500 mg/m^2^, after four weeks 750 mg/m^2^, followed by 1000 mg/m^2^ every four weeks for a total of six infusions. Furthermore, oral tacrolimus was initiated. After the six pulses of cyclophosphamide, rituximab was administered for remission maintenance. Thus, P7 is currently treated with hydroxychloroquine, tacrolimus, low dose prednisone and rituximab.

In patient 8 (P8), acute myeloid leukemia was diagnosed first. After induction therapy allogeneic stem cell transplantation was performed. Graft-versus-host disease occurred in the intestinal tract. Subsequently, P8 developed myalgia, arthralgia and thoracic pain. In the laboratory measurements, MDA5 antibodies were found. MRI of the upper arm was performed with diagnosis of myositis. Due to this findings including a restriction in the lung function testing, MDA5 CADM was diagnosed without typical CT morphological findings of the lung. Oral tacrolimus was recommended but fearing a leukemic relapse, P8 did not follow that advice. At the present time, MDA5 antibody is negative in P8. Watch and wait as therapy strategy seems possible, but regular lung function testing is still crucial to identify a potential disease progression.

## Discussion

MDA5-positive amyopathic DM is a rare form of DM in Europe. Since this DM subtype is more common in Asians, data predominantly refers to Asian cases. There is still limited data about treatment of MDA5-positive amyopathic DM.

There is an increasing number of ICI-related myositis (Immune Checkpoint Inhibitor-Related Myositis) due to a rising number of patients receiving immune checkpoint inhibitors for cancer treatment. The muscle inflammatory involvement arises from a different origin and then MDA5-positive amyopathic DM [10].

There is a general consensus that glucocorticoid therapy improves strength and preserves muscle function in DM, polymyositis and ICI-related myositis [9, 13].

Depending on the activity of disease, methylprednisolone pulse (500 mg-1000 mg per day for three days) or oral prednisone (1 mg/kg) per day with tapering schedule is needed. However, 50 percent of patients with myositis do not respond to glucocorticoid therapy alone [14]. In these cases, an alternative diagnosis should be considered. Differential diagnosis include glucocorticoid-induced myopathy or an unrecognized malignancy leading to paraneoplastic myositis. Most malignancies are diagnosed within a two-year period before and after the development of myositis [15].

Azathioprine or methotrexate is currently first-line glucocorticoid-sparing agents in DM. Patients who are severely ill may benefit from intravenous immunoglobulins as well [4, 16]. There are various therapeutic options for patients who do not respond to glucocorticoids and azathioprine/methotrexate. But neither of these options have been widely studied in clinical trials nor have they been evaluated for the rare amyopathic subtype described in this case series. They mostly refer to small case studies. Prior publications showed that refractory myositis can be treated effectively with rituximab [8].

Other treatment strategies include calcineurin inhibitors, mycophenolate mofetil, cyclophosphamide and JAK inhibitors. Comparative studies between these strategies have not been performed yet.

Due to the lack of severe manifestations of myositis and the highly complex clinical phenotypes, there is often a delay in diagnosis of MDA5-DM. Our cohort is no exception: None of our patients presented with an increased creatine kinase (CK).

Three of the eight cases (P3, P6, P7) were initially misdiagnosed with another inflammatory disease.

Since a rapidly progressive pulmonary involvement in MDA5-positive amyopathic DM may occur, the prompt initiation of therapy is essential to improve disease specific morbidity and mortality. Due to the high mortality of 80% [2], all patients received initially an intravenous prednisone pulse or oral prednisone.

Due to their pulmonary involvement, cyclophosphamide and oral tacrolimus were additionally initiated in four patients shortly after the diagnosis of CADM was made. There are some case series recommending this `triple therapy` at the onset of disease with good outcome [5, 7], and indeed, all or our patients are still alive. Depending on the first presentation of the disease, we also performed plasmapheresis, intravenous prostaglandin and intravenous immunoglobulin administration in two patients, respectively.

No changes in remission maintenance therapy were necessary so far due to stable disease (Table 4). Although the treatment regimens have not been standardized in our cohort of eight patients, it shows that early and aggressive immunosuppressive combination treatment can induce a stable remission in patients with MDA5-positive amyopathic DM.

Over this follow-up period, two malignancies were reported in a temporal context to the diagnosis of MDA5 amyopathic DM: P1 had concurrent diagnosis of non-Hodgkin lymphoma, in P8 acute myeloid leukemia was diagnosed prior to CAMD.

Since this entity is rare in Europe, increasing awareness for the clinical features of amyopathic DM is crucial to decrease disease-related mortality and long-term disability. It is although important to differ from the ICI-related myositis seen in patients receiving immune checkpoint inhibitors for cancer treatment.

Given the association of both conditions with malignancies, further research into the underlying pathogenesis and shared mechanisms could provide insights in a maybe common molecular or genetic pathway. Furthermore, discovery of specific biomarkers could help differentiate between the two conditions and advanced imaging modalities could help identify unique patterns of muscle involvement in each condition.

However, our study has some limitations. First of all, it is a single center study which may limit the generalizability of the data. Second, we analyzed retrospective data which often comes with incomplete data collections or a selection bias. Future prospective studies should be planned with a multicenter approach and a longer study duration with implemented standardized protocols for data collection. Additional studies as well on long-term outcomes, especially post-therapy, as epidemiological studies to understand the prevalence of coexistence would be beneficial.

## Data Availability

The data that support the findings of this study are available from the corresponding author (TT) upon reasonable request.
